# Inhibitory effects of an aqueous extract from Cortex Phellodendri on the growth and replication of broad-spectrum of viruses in vitro and in vivo

**DOI:** 10.1186/s12906-016-1206-x

**Published:** 2016-08-02

**Authors:** Jae-Hoon Kim, Prasanna Weeratunga, Myun Soo Kim, Chamilani Nikapitiya, Byeong-Hoon Lee, Md Bashir Uddin, Tae-Hwan Kim, Ji-Eun Yoon, Chung Park, Jin Yeul Ma, Hongik Kim, Jong-Soo Lee

**Affiliations:** 1 College of Veterinary Medicine, Chungnam National University, 220 Gung-Dong, Yuseong-Gu, Daejeon 305-764 Republic of Korea; 2 Vitabio, Inc., Daejeon, 305-764 Republic of Korea; 3 Sylhet Agricultural University, Sylhet, Bangladesh; 4 Korean Medicine (KM) Based Herbal Drug Development Group, Korea Institute of Oriental Medicine, Deajeon, 305-764 Republic of Korea; 5 LINC Project Group, Daejeon University, 62 Daehak-Ro, Dong-Gu, Daejeon 300-716 Republic of Korea

**Keywords:** Cortex Phellodendri, Herbal medicine, Berberine, Anti-viral effect

## Abstract

**Background:**

Cortex Phellodendri (C. Phellodendri), the dried trunk bark of *Phellodendron amurense* Ruprecht, has been known as a traditional herbal medicine, showing several bioactivities. However, antiviral activity of C. Phellodendri aqueous extract (CP) not reported in detail, particularly aiming the prophylactic effectiveness.

**Methods:**

In vitro CP antiviral activity evaluated against Influenza A virus (PR8), Vesicular Stomatitis Virus (VSV), Newcastle Disease Virus (NDV), Herpes Simplex Virus (HSV), Coxsackie Virus (H3-GFP) and Enterovirus-71 (EV-71) infection on immune (RAW264.7) and epithelial (HEK293T/HeLa) cells. Such antiviral effects were explained by the induction of antiviral state which was determined by phosphorylation of signal molecules, secretion of IFNs and cytokines, and cellular antiviral mRNA expression. Furthermore, Compounds present in the aqueous fractions confirmed by HPLC analysis and evaluated their anti-viral activities. Additionally, in vivo protective effect of CP against divergent influenza A subtypes was determined in a BALB/c mouse infection model.

**Results:**

An effective dose of CP significantly reduced the virus replication both in immune and epithelial cells. Mechanically, CP induced mRNA expression of anti-viral genes and cytokine secretion in both RAW264.7 and HEK293T cells. Furthermore, the main compound identified was berberine, and shows promising antiviral properties similar to CP. Finally, BALB/c mice treated with CP displayed higher protection levels against lethal doses of highly pathogenic influenza A subtypes (H1N1, H5N2, H7N3 and H9N2).

**Conclusion:**

CP including berberine play an immunomodulatory role with broad spectrum antiviral activity, due to induction of antiviral state via type I IFN stimulation mechanism. Consequently, C. Phellodendri could be a potential source for promising natural antivirals or to design other antiviral agents for animal and humans.

**Electronic supplementary material:**

The online version of this article (doi:10.1186/s12906-016-1206-x) contains supplementary material, which is available to authorized users.

## Background

Several viruses cause malignant diseases worldwide, resulting in significant mortality and economic losses. For instance, Influenza A viruses are responsible for seasonal epidemics and have caused three pandemics in the 20th century (1918, 1957, and 1968) [1]. In addition, viral diseases cause serious threats to livestock sector and animal welfare together with productivity losses, uncertain food security and negative impacts on human health [2].

Although various prophylactic or therapeutic drugs and vaccines have been developed to prevent and treat viral diseases, the emergence of novel mutants or resistant virus strains reduces their efficacy and lead to the public health problems. Especially, with a view to provide effective methods for preventing viral diseases, researches have been attempted to identify novel materials with antiviral activities from natural or synthetic resources.

Natural products are one of relevant sources for antiviral substances. Therefore, many screening efforts have been made to identify antiviral substances from natural sources with high efficacy, low toxicity and minor side effects. Hence, treatments with herbal extracts having antiviral activities may assist to recover many animals through effective feed conversions and weight gains to result better economic returns. Consequently, as an alternative to conventional chemical agents, a large number of phytochemicals which containing extracts or pure substances from medical plants have been recognized as antivirals [3].

To identify potential antiviral agents, 200 natural oriental herbal medicines were screened and among them, Cortex Phellodendri (C. Phellodendri) found to display a wide array of antiviral activities. C. Phellodendri (Huangbai in Chinese), the dried bark of *Phellodendron amurense* Ruprecht (Family: Rutaceae), is one of the 50 fundamental herbs of traditional Chinese medicine and has been used against osteoarthritis, weight loss, obesity, diarrhea, diabetes, pneumonia and eye infections for a long period of time [4]. This herb is widely found in China and the Korean peninsula, and a wide range of primary scientific articles are already available on the activities of extracts of Phellodendron bark, although the underline mechanisms in the therapeutic process remain unclear. Further, the antiviral activity of C. Phellodendri has not been scientifically described.

In this study, the antiviral activities of C. Phellodendri aqueous extracts (CP) against wide array of viruses in vitro and in vivo were evaluated*.* Additionally, the immune-modulatory potential of C. Phellodendri which regulates the antiviral immune response was confirmed.

## Methods

### Cells and viruses

RAW264.7 (ATCC® TIB-71™), HEK293T (ATCC® CRL-11268™), HeLa (ATCC® CCL-2™) and MDCK (ATCC CCL-34, NBL-2) cells were maintained in Dulbecco’s Modified Eagle’s Medium (DMEM) (Invitrogen, Carlsbad, CA, USA) supplemented with 10 % fetal bovine serum (FBS) (Gibco, Grand Island, NY, USA) and 1 % antibiotic-antimycotic solution (Gibco, Grand Island, NY, USA) at 37 °C with 5 % CO_2._ Green Fluorescent Protein (GFP)-tagged Influenza A (A/PuertoRico/8/34(H1N1) (PR8-GFP), Newcastle Disease Virus (NDV-GFP) and challenge viruses of influenza A subtypes [{A/Aquaticbird/Korea/W81/2005 (H5N2)}, {A/PR/8 /34(H1N1)}, {A/Aquaticbird/Korea/W44/2005(H7N3)} and {A/Chicken /Korea/116/2004(H9N2)}] were propagated in the allantoic fluid of 10-day-old chicken embryos. Vesicular Stomatitis Virus (VSV-GFP), Herpes Simplex Virus (HSV-GFP) and Enterovirus-71 (EV-71) were propagated on confluent Vero cells (ATCC® CCL-81™) and Coxsackie virus (H3-GFP) was propagated on confluent A549 (ATCC® CCL-185™) cells.

### Plant materials and total aqueous extract preparation

Crude plant material, the dried bark of *Phellodendron amurense* Ruprecht (specimen number: *Phellodendron amurense* Rupr., PSNA200005151048; Department of Pharmacology at Busan National University, Korea), was purchased from Jaecheon Oriental Herbal Market (Jaecheon, Korea) and verified by Professor Ki-Hwan Bae at the College of Pharmacy, Chungnam National University. Water-soluble herbal extract of C. Phellodendri was prepared by Vitabio Corporation (Daejeon, Korea).

### Determination of Effective Concentration (EC_50_) of Cortex Phellodendri in vitro

The EC_50_ can be defined as the observed extract concentration at which 50 % reduction in virus titer. To determine the EC_50_ values of CP against divergent viruses in vitro, a modified GFP assay was developed using RAW264.7, HEK293T and HeLa cell lines [5]. Raw264.7, HEK293T and HeLa cells were cultured in 96-well plates and after 12 h of incubation, the media was replaced with 2-fold serially diluted CP from original stock (0.1 mg/ml). At 12 h post-treatment (hpt), RAW264.7 cells were infected with PR8-GFP (MOI = 1.0), VSV-GFP (MOI = 1.0) or NDV-GFP (MOI = 3.0); HEK293T cells with VSV-GFP (MOI = 0.2) or HSV-GFP (MOI = 2.0); and HeLa cells with H3-GFP (MOI = 3.0) or EV-71 (MOI = 0.5) using DMEM containing 1 % FBS. At 2 h post-infection (hpi), the inocula were replaced with DMEM (10 % FBS). GFP expression was measured at 24 hpi using Glomax multi-detection system (Promega, WI, USA). EC_50_ values were calculated as the extract concentration which yielded 50 % GFP expression or in the EV-71, 50 % reduction in viral cytopathic effects (CPE).

### Cytotoxicity assay (CC_50_) of Cortex Phellodendri in vitro

The CC_50_ assay was performed in 72-well tissue culture plates and the CC_50_ was determined through trypan blue exclusion test as described previously [6]. Increasing concentrations (0.1-16 μg/ml) of the extract were added to 75–80 % confluent RAW264.7, HEK293T and HeLa cell monolayers. After 24 h, the cell viability of each treatment group was determined by trypan blue staining. Concentrations of the extract plotted against the cell viabilities, and CC_50_ was calculated as the concentration of the extract resulting 50 % cell viability.

### Antiviral assays in Cortex Phellodendri-treated RAW264.7 and epithelial (HEK293T or HeLa) cells

Virus replication inhibition assay was performed using the GFP viruses as described previously with some modifications [7]. RAW264.7 (12-well plates; 8 x 10^5^ cells/well), HEK293T and HeLa cells (6-well plates; 1 x 10^6^ cells/well) were incubated at 37 °C for 12 h. For the pre-treatment assay, DMEM alone (untreated and virus-only groups), positive control; DMEM with 1000 U recombinant mouse or human interferon-β (rmIFN-β or rhIFN-β, Sigma, St. Louis, USA) and DMEM with 1.0 μg/ml of CP were added to the cells. At 12 hpt, RAW264.7 cells were infected with either PR8-GFP (MOI = 1.0), NDV-GFP (MOI = 3.0) or VSV-GFP (MOI = 1.0) using DMEM supplemented with 1 % FBS. HEK293T cells were infected with VSV-GFP (MOI = 0.2) or HSV-GFP (MOI = 2.0). HeLa cells were infected with H3-GFP (MOI = 3.0) or EV-71 (MOI = 0.5). At 2 hpi, wells were gently washed with PBS and replaced the media with DMEM (10 % FBS). GFP expression or CPE, which reflects virus replication, was observed at 12 or 24 hpi under 200X magnification. The cell viability and the virus titer were determined at 12 or 24 hpi. GFP expression was measured at 24 hpi using Glomax multi-detection system (Promega, Wisconsin, USA) according to the manufacturer’s instructions.

### NDV-GFP mRNA expression in RAW 264.7 cells

Total mRNA from RAW264.7 cells infected with NDV-GFP (MOI = 3.0) was extracted using the RNeasy Mini Kit (Qiagen, Seoul, Korea) and cDNA was synthesized using reverse transcriptase (Toyobo, Japan) according to manufacturer’s protocol. Real-time polymerase chain reaction (RT-PCR) was performed using gene specific primers to determine the NDV-GFP mRNA expression levels. For the NDV Matrix (M) gene, forward primer was 5′-TCGAGICTGTACAATCTTGC-3′ and the reverse primer was 5′- GTCCGAGCACATCACTGAGC-3′ [8]. For the GAPDH (housekeeping gene), the forward primer was 5′-TGACCACAGTCCATG CCATC-3′ and the reverse primer was 5′-GACGGACACATTGGG GGTAG-3′ [9]. Equal amount of PCR products were run on 1.5 % ethidium bromide agarose gels and visualized using GelDoc Imaging System (Bio-Rad, Seoul, Korea). The relative band intensity (RBI) of the M gene was compared with GAPDH using Band Quantification Software (Bio-Rad, Seoul, Korea).

### Virus titration from Cortex Phellodendri-treated cell supernatants or cells

Viral titers were determined with cell supernatants (VSV-GFP and H3-GFP) or cells (PR8-GFP and HSV-GFP) in Vero cells using a standard plaque assay. EV-71 titer measured by the 50 % tissue culture infectious doses (TCID_50_) using HeLa cells with some modifications. Briefly, 75–80 % confluent HeLa cells grown in 96-well micro-titer plates were infected with 10-fold serial diluted supernatants (50 μl/well) collected from the infected cells. At 2 hpi, DMEM (10 % FBS) containing L-1-tosylamido-2-phenylethyl chloromethyl ketone (TPCK) trypsin (Thermo Fisher Scientific, Rockford, USA) was added to the infected wells and incubated for another 2 days. CPE were observed daily under 200X magnification, and the titers were determined by TCID_50_.

### Oral administration of Cortex Phellodendri and virus challenge in BALB/c mice

Five-week-old 52 BALB/c mice (16 ± 1 g) were used to conduct in vivo experiments (Table 1) For the survival rate analysis, 40 mice were grouped in to 4 main groups (*n* = 10), each containing 2 subgroups, control (*n* = 5) and CP treated group (*n* = 5). For the lung virus titration another 12 mice were grouped into 2 sub-groups, control (*n* = 6) and CP treated group (*n* = 6), and lung tissue was extracted at 3 (*n* = 3) and 5 dpi (*n* = 3) from each control and treated group. CP were orally administered to the mice (0.8 μg/g body weight) in a total volume of 200 μl at 1, 3 and 5 days before infection. PBS (200 μl) was orally administered to control groups mice. Treatment and challenge experiments were conducted in an approved BSL-2^+^ laboratory facility. Mice were intranasally infected with 5 times 50 % minimum lethal dose (MLD_50_) of H1N1, H5N2, H7N3 or H9N2 in 20 μl of PBS per mouse. Body weight and survival were monitored up to 13 dpi. Mice exhibiting more than 25 % body weight loss were considered as an experimental end point and were humanely killed. Animal study was conducted under appropriate conditions with the approval of the Institutional Animal Care and Use Committee of Bioleaders Corporation, Daejeon, South Korea, Protocol number: BSL-ABLS-13-008.

**Table 1:** Mouse groups of in vivo experiments

Virus	Group	Treatment	Extract Dose (μg/g body weight)	Experiment
H1N1	Control (*n* = 5)	PBS	-	Survival rate
	CP (*n* = 5)	CP	0.8	Survival rate
H5N2	Control (*n* = 5)	PBS	-	Survival rate
	CP (*n* = 5)	CP	0.8	Survival rate
H7N3	Control (*n* = 5)	PBS	-	Survival rate
	CP (*n* = 5)	CP	0.8	Survival rate
H9N2	Control (*n* = 5)	PBS	-	Survival rate
	CP (*n* = 5)	CP	0.8	Survival rate
H1N1	Control (*n* = 6) 3 dpi (*n* = 3), 5 dpi (*n* = 3)	PBS	-	Lung titration
	CP (*n* = 6) 3 dpi (*n* = 3), 5 dpi (*n* = 3)	CP	0.8	Lung titration

### Determination of lung viral titers

Lung tissues from euthanized mice were collected aseptically, and lung viral titer was calculated by the Reed and Muench method and expressed as Log_10_ TCID_50_/Lung. In the modified hemagglutination assay (HA), instead of turkey red blood cells (RBC), 0.5 % chicken RBC was used and wells containing HA were scored as positive [10].

### Detection of IFN-β and pro-inflammatory cytokines

Cells were treated with 1000 units/ml rmIFN-β or rhIFN-β and 1.0 μg/ml CP in DMEM supplemented with 10 % FBS or media alone, and then incubated at 37 °C with 5 % CO_2_. The supernatants were harvested at 0, 12 and 24 hpt. Using commercial ELISA kits, murine interleukin (IL)-6, IL-12, tumor necrosis factor-alpha (TNF-α) (BD Bioscience, USA), murine IFN-β (PBL Interferon Source, USA), human IFN-β (TFB Inc., Tokyo, Japan) and human IL-6 (Invitrogen, Carlsbad, California, USA) were measured according to manufacturer’s protocol.

### Determination of the effects of Cortex Phellodendri on Type I IFN-related protein phosphorylation by Immunoblot analysis

RAW264.7 cells were treated with DMEM alone (negative control), DMEM with 100 ng/ml LPS (positive control), or DMEM with 1.0 μg/ml CP, and cells were harvested at 0, 3, 6, 12, and 24 hpt. Cell pellets were washed with PBS and whole cell lysates were subjected to SDS-PAGE followed by standard immunoblotting with indicated antibodies: anti-IRF3 (Abcam, #ab25950), anti-phospho-IRF3 (Ser396) (Cell Signaling, #4947), anti-p65 (Cell Signaling, #4764S), anti-phospho-p65 (Cell Signaling, #3031S), anti-STAT1 (Cell Signaling, #9175), anti-phospho-STAT1 (Cell Signaling, #9167), anti-TBK1 (Cell Signaling, #3504S), or anti-phospho-TBK1 (Cell Signaling, #5483S), anti-p38 (Cell Signaling, #9212), anti-phospho-p38 (Cell Signaling #4631S), anti-ERK (Cell Signaling, #9102), anti-phospho-ERK (Cell Signaling, #9102S), or anti-β-actin (Santa Cruz SC 47778).

### Level of mRNA induction by Cortex Phellodendri in vitro

RAW264.7 or HEK293T cells were treated with DMEM supplemented with media alone (negative control), DMEM with 1000 units/ml rmIFN-β or rhIFN-β (positive control), or DMEM with 1.0 μg/ml CP and the cells were harvested at 0, 3, 6, 12, and 24 hpt. The total RNA isolation, cDNA synthesis, and visualization of band intensity after PCR were as previously described. Additionally, different cDNA levels from HEK293T cells were measured by quantitative RT- PCR (qRT-PCR) using a QuantiTech SYBR Green PCR kit (Qiagen, Seoul, Korea) on a Mygenie96 thermal block (Bioneer, Daejeon, Korea). The PCR primers are listed in Tables 3 and 4.

### HPLC analysis for chemical characterization of aqueous plant extract of Cortex Phellodendri

The liquid chromatography-mass spectrometry (LC-MS) analysis was performed on an Agilent 1200 Series high-performance liquid chromatography (HPLC) (Agilenttechnologies CO, USA) connected to a linear ion trap mass spectrometer 4000 QTRAP system (AB Sciex CO., Canada) equipped with an ESI TurboV source. Chromatography was carried out on a column ZORBAX Eclipse XDB-C18 (4.6 x 150 mm, I.D.-5 μm) (Agilent technologies CO., USA). The mobile phase consisted of 1 % Formic acid (Solvent A) and Acetonitrile (Solvent B) in the gradient mode as follows : 0–8 min 0–25 % B; 8–12 min 25–35 % B; 12–17 min 35–50 % B; 17–25 min 50–100 % B; 25–28 min 100–0 % B at flow rate of 1.0 ml/min at 30 °C. Conditions of the MS analysis were as follows: positive ion mode, spectra range from m/z 100 to 500, nebulizer; 70.0 psi. Molecular weight identification of purified fraction (PA-6) and berberine was carried out by electrospray ionization mass spectrometry (ESI-MS).

### Determination of antiviral characteristics of berberine

PA-6 antiviral activity and cytokine inducing ability was further tested in immune (RAW264.7) cells using an effective dose (10.0 μg/ml). Inhibition of virus replication (PR8-GFP) in RAW264.7 cells, virus titration and induction of cytokine secretion were monitored according to the protocols described above sections.

### Statistical analysis

Statistical analysis was performed using GraphPad Prism software version 6 for Windows (GraphPad Software, USA). Data are presented as the means ± standard deviations (S.D.) and are representative of at least three independent experiments. Differences between groups were analyzed by analysis of variance (ANOVA). Unpaired *t*-test was performed at each time points to compare the control and NDV infected extract-treated groups. Results for percent initial body weight were also compared by Student’s *t* test. Comparison of survival was done by log-rank test using GraphPad Prism 6 version. *P* < 0.05 were regarded as significant.

## Results

### Inhibition of viral replication in Cortex Phellodendri-treated RAW264.7 cells

**Fig. 1:**
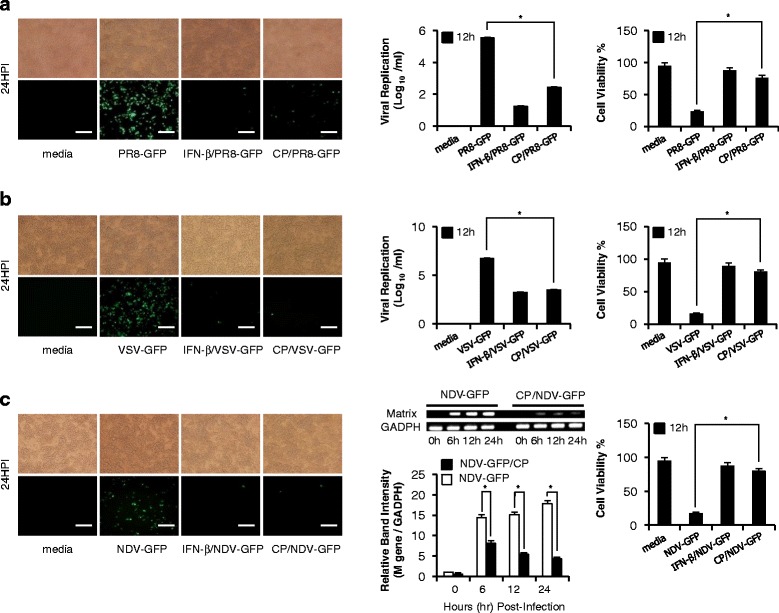
Cortex Phellodendri antiviral activities in RAW264.7 cells. RAW264.7 cells treated with media alone, 1.0 μg/ml CP, or 1000 unit/ml rmIFN-β at 12 h prior to infection with **a** PR8-GFP or **b** VSV-GFP or **c** NDV-GFP at 1.0 MOI. Images were obtained at 12 hpi (200X magnification). Cell viabilities were determined by trypan blue exclusion and presented as a percentage of the control (cells without treatment). The PR8-GFP and VSV-GFP titer were determined by standard plaque assay. NDV M-gene mRNA expression was evaluated using gene specific PCR primers and all the samples normalized using GAPDH. Cell viabilities are expressed as mean ± SD. Error bars indicate the range of values obtained from counting in triplicate in three independent experiments (*P < 0.05 indicates a significant difference between groups)

In vitro antiviral effects of CP were evaluated by observing the amount of GFP expressed in the infected cell to analyze the effect on RNA and DNA viral replications. RAW264.7 cells treated with CP exhibited a marked reduction in GFP expression as compared to the untreated groups against PR8-GFP (Fig. 1a), VSV-GFP (Fig. 1b) and NDV-GFP (Fig. 1c). GFP expression was significantly reduced in extract-treated cells compared to the untreated groups (data not shown), which correlated with viral titers. CP treatment reduced the viral titers by 2.3- and 2-fold against PR8-GFP and VSV-GFP, respectively (Fig. 1a and b). Importantly, CP-treated cells had ≥ 70 % cell viability within 24 hpi for all tested viruses compared to untreated cells which had significantly higher cell death following virus infection (Fig. 1a-c). M-gene mRNA expression which represent the NDV virus replication, decreased time coarsely in extract treated cells compared to untreated groups (Fig. 1c right panel). These results indicate that the CP has potential to inhibit VSV-GFP, PR8-GFP and NDV-GFP replication in RAW264.7 cells.

### Inhibition of viral replication in Cortex Phellodendri-treated HEK293T and HeLa cells

**Fig. 2:**
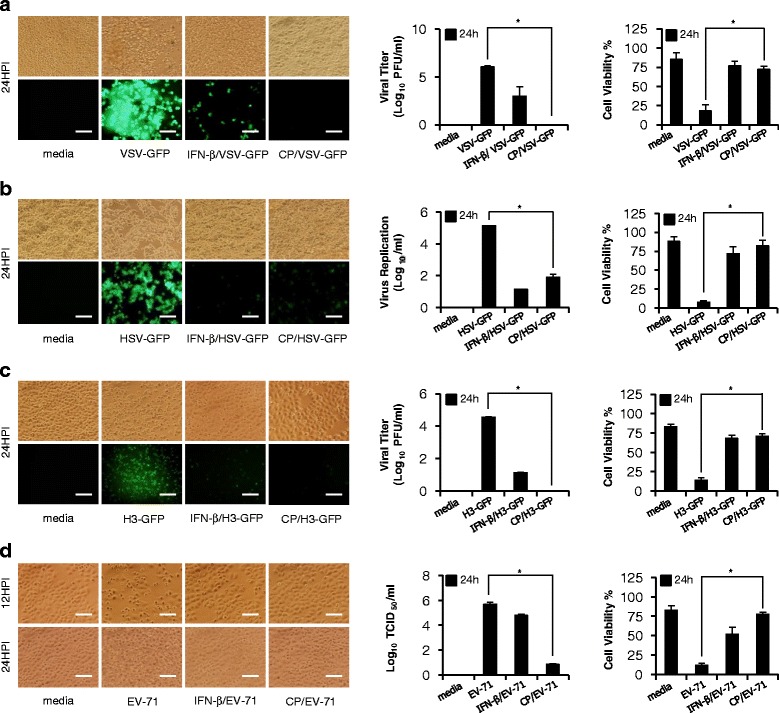
Cortex Phellodendri antiviral activities in HEK293T and HeLa cells. HEK293T and HeLa cells treated with media alone, 1.0 μg/ml CP, or 1000 unit/ml rhIFN-β at 12 h prior to infection with **a** VSV-GFP or **b** HSV-GFP or **c** H3-GFP or **d** EV-71 at 1.0, 1.0, 3.0 and 0.5 MOI, respectively. Images were obtained at 24 hpi (200X magnification). The tests were performed in triplicate. Virus titrations are expressed as mean ± SD. Error bars indicate the range of values obtained from two independent experiments. Cell viabilities were determined by trypan blue exclusion, presented as a percentage of the control (cells without treatment). (*P < 0.05 indicates a significant difference between groups)

CP antiviral activity on non-immune epithelial cell lines such as HEK293T and HeLa cells was determined. Upon pre-treatment of the CP, HEK293T cells displayed marked reduction in GFP expression and viral titers by 6- and 2.6-fold against VSV-GFP and HSV-GFP, respectively, at 24 hpi compared to untreated groups (Fig. 2a and b). Moreover, CP antiviral activity in HeLa cells against H3-GFP and EV-71 was determined. CP pre-treatment exhibited markedly reduced H3-GFP expression and reduced viral titers by 4.5-fold at 24 hpi together with significant reduction in cell death in the extract treated group compared to untreated group (Fig. 2c). Furthermore, EV-71 induced CPE were markedly inhibited and upon pre-treatment with the extract, a low level of viral replication was observed (Fig. 2d). Collectively, it is also clear that the CP has ability to suppress DNA or RNA viral replication in epithelial cell lines.

### EC_50_ and CC_50_ of Cortex Phellodendri extracts in vitro

As shown in Table 2, CP inhibited the replication of PR8-GFP (MOI = 1.0), VSV-GFP (MOI = 1.0) and NDV-GFP (MOI = 3.0) by 50 % at an EC_50_ of 0.89 ± 0.09 μg/ml, 0.71 ± 0.15 μg/ml and 0.57 ± 0.13 μg/ml, respectively in RAW264.7 cells. Moreover, the aqueous extract inhibited VSV-GFP (MOI = 0.2) and HSV-GFP (MOI = 2.0) replication by 50 %, showing EC_50_ of 0.63 ± 0.06 μg/ml and 0.83 ± 0.07 μg/ml, respectively in HEK293T cells. Additionally, observed EC_50_ against H3-GFP and EV-71 were 0.75 ± 0.12 μg/ml and 0.39 ± 0.07 μg/ml, respectively in HeLa cells. According to the EC_50_ and considering its effectiveness and convenience during the experiments, 1.0 μg/ml of CP were selected as the optimum dosage for further in vitro antiviral assays.

**Table 2:** Determination of EC_50_ and CC_50_ of Cortex phellodendri in RAW264.7, HEK293T and HeLa cells

Cell	Ec_50_ ± S.D.^b^(μg/ml)						CC_50_ ± S.D.^c^(μg/ml)
	PR8-GFP	VSV-GFP	NDV-GFP	H3-GFP	HSV-GFP	EV-71	
Raw264.7	0.89 ± 0.09	0.71 ± 0.15	0.57 ± 0.13	-	-	-	7.31 ± 0.12
HEK293T	-	0.63 ± 0.06	-	-	0.83 ± 0.07	-	6.36 ± 0.06
HeLa	-	-	-	0.75 ± 0.12	-	0.39 ± 0.07^a^	8.06 ± 0.04

^a^Effective concentration for 50 % reduction in CPE

^b^Effective concentration for 50 % reduction in GFP expression

^c^Cytotoxic concentration causing 50 % cell death. The results are a mean of three independent experiments

CP had CC_50_ of 7.31 ± 0.12 μg/ml, 6.36 ± 0.06 μg/ml and 8.06 ± 0.04 μg/ml in Raw264.7, HEK293T and HeLa cells, respectively (Table 2). Importantly, CC_50_ of CP were several magnitudes higher than the EC_50_ of tested viruses in their respective cell lines. Selection indexes (SI) of CP for PR8, VSV-GFP and NDV-GFP in RAW264.7 cells were 8.2, 10.2 and 12.8, respectively; for VSV-GFP and HSV-GFP in HEK293T cells were 10.1 and 7.7, respectively; and for H3-GFP and EV-71 in HeLa cells were 10.7 and 20.9, respectively. These results suggest that CP is relatively non-toxic and could be expedient as a wide prophylactic and/or therapeutic potentials.

### Protection against influenza A virus infection by oral administration of Cortex Phellodendri in Balb/c mice

**Fig. 3:**
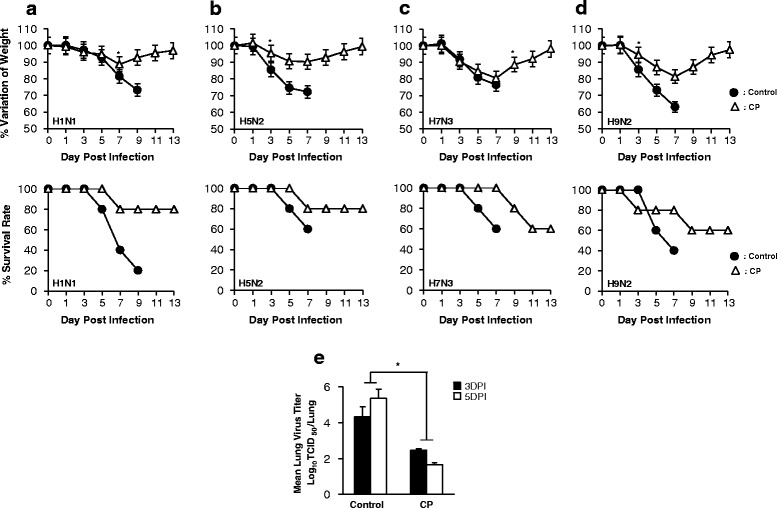
Oral administration of Cortex Phellodendri provides protection against lethal infection with divergent influenza A subtype in BALB/c mice. CP were orally administered to 5-week-old female BALB/c mice (0.8 μg/g body weight) in a total volume of 200 μl at 1, 3 and 5 days before infection with 5MLD_50_ **a** H1N1, **b** H5N2, **c** H7N3 and **d** H9N2 Influenza A subtypes. PBS (200 μl) was orally administered to control groups. Percent survival and weight changes after challenge were recorded until 13 dpi. **e** Virus titers in lung tissues of H1N1 infected mice were measured by TCID_50_ at 3 and 5 dpi. (*P < 0.05 indicates a significant difference between groups)

Minimum effective dose (0.8 μg/g body weight) was chosen based on our previous in vivo experimental experiences with various other herbal extracts (Data not shown), and groups of BALB/c mice were infected with lethal doses (5MLD_50_) of H1N1, H5N2, H7N3 and H9N2. Around 4 dpi, most of the control group mice exhibited severe clinical signs of respiratory disease. Additionally, mice showed obvious clinical signs, including decreased activity, huddling, hunched posture, and ruffled fur. Further, body weights were decreased progressively and the control groups succumbed to death by 9 dpi for all of the viruses tested. Strikingly, CP-treated mice showed ≤ 20 % body weight loss between 5 and 7 dpi and began to recover their lost weight by 8 dpi, returning to their normal state by 13 dpi (Fig. 3). All the groups that were orally inoculated with the herbal extract had higher survival rates (80 % survival for H1N1 and H5N2 and 60 % survival for H7N3 and H9N2) than control group. Survived mice in these groups did not show obvious clinical signs except slight weight losses.

Lung tissues were collected from the H1N1-infected experimental group to examine the ability of CP to inhibit viral replication in the lung. In the control group, H1N1 replicated efficiently with viral titer of 4.19 and 5.34 log TCID_50_/Lung at 3 dpi and 5 dpi, respectively. In comparison, the viral burdens in CP treated groups were several folds lower than control group which had 2.3 and 1.9 log TCID_50_/Lung, at 3 dpi and 5 dpi, respectively (Fig. 3e). These findings suggest that CP can induce sufficient inhibition of viral replication in lung and promoted the survival of mice against lethal infections of diverse influenza A viruses.

### Cortex Phellodendri extracts induced the secretion of IFN-β or pro-inflammatory cytokine and activation of Type I IFN signal molecules

**Fig. 4:**
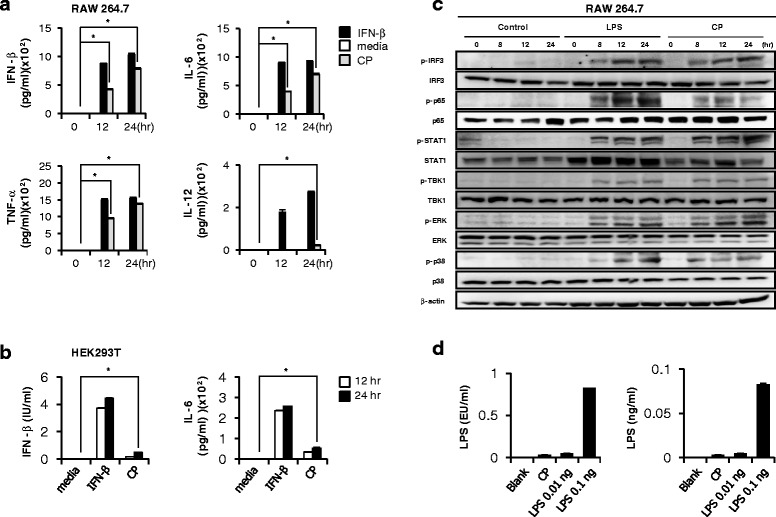
Induction of cytokines and the phosphorylation of signal molecules present in the type I IFN pathway by Cortex Phellodendri in vitro. **a** RAW264.7 and **b** HEK293T cells were treated with media alone, with 1000 unit/ml rmIFN-β or rhIFN-β, or with 1.0 μg/ml CP. IFN-β, IL-6, TNF-α and IL-12 were measured by ELISA at 0, 12 and 24 hpt. The tests were performed in triplicate. The data shows representative means ± SD. (*P < 0.05 indicates a significant difference between groups). **c** Cells treated with LPS or CP were harvested at 0, 8, 12, and 24 hpt and subjected to standard immunoblot analysis for type I IFN-related or NF-kB related protein phosphorylation. **d** Detection of contaminated endotoxin in CP by Limulus Amebocyte Lysate (LAL) assay. (EU; endotoxin unit)

To determine the effect of CP on inhibition of the viral replication, the levels of Interferon-β (IFN-β) and pro-inflammatory cytokines were measured in the CP-treated cell supernatant of RAW264.7 and HEK293T cells (Fig. 4). CP (1.0 μg/ml) induced high levels of secreted TNF-α, IL-6, IL-12 and IFN-β, similar to the pattern observed in IFN-β treated cells in RAW264.7 cells (Fig. 4a). Though IL-6 and IFN-β secretion levels were lower than IFN-β treated cells, CP-treated HEK293T cells secreted detectable levels of IL-6 and IFN-β (Fig. 4b). Consequently, these data indicated that CP can mediate the antiviral responses in immune or epithelial cells by triggering the expression of IFNs and pro-inflammatory cytokines which may stimulate the antiviral state in cells, playing a crucial role in inhibiting viral replication.

Phosphorylation levels of IFN related signal molecules and NF-kB activation related molecule (p65) were measured in CP-treated RAW264.7 cells to determine the type I IFN signal transduction. As shown in Fig. 4c, CP significantly up-regulated the IRF-3, STAT1, TBK1, p65, p38 and ERK phosphorylation, the main signaling molecules in the type I IFN and NF-kB pathways. Especially the phosphorylation of IRF3, the key indicator of IFN signal transduction was induced at 8 hpt, and this effect markedly increased with the time (Fig. 4c). Furthermore, increased STAT-1 phosphorylation indicates the transcriptional activation of interferon-stimulating genes (ISGs) which involved in controlling viral infection. Additionally, CP-treated RAW264.7 cells obviously activated NF-kB (p65), leading to strong secretion of pro-inflammatory cytokines. CP was tested with LPS (positive control) using a Limulus Amebocyte Lysate (LAL) assay. Only a trace amount of endotoxin was found in CP (Fig. 4d), thus confirmed that the induction of type I IFN and pro-inflammatory signaling was not due to LPS stimulation.

### Induction of antiviral gene expression by Cortex Phellodendri

**Fig. 5:**
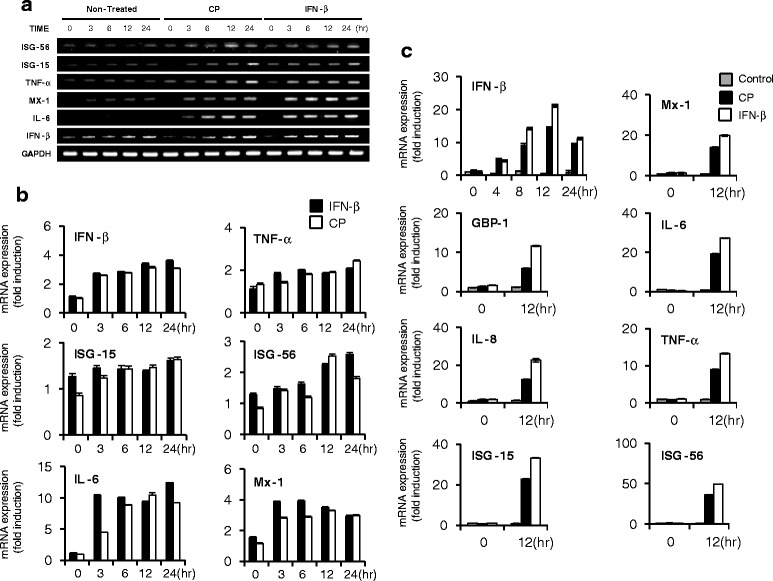
Induction of IFN-β, IFN-related and antiviral genes by Cortex Phellodendri in vitro*.* RAW264.7 and HEK293T cells were treated with media alone, CP (1.0 μg/ml), or 1000 units/ml of rmIFN-β or rhIFN-β. Time-dependent changes in mRNA expression after treatment in **a** RAW264.7 and **c** HEK293T cells were confirmed by RT-PCR and qRT-PCR respectively. **b** RBI of IFN-β, IFN-α, TNF-α, IL-6, and IFN-related genes (Mx1, ISG15 and ISG56) were determined using the image analysis program and are shown as a bar graph. All the samples normalized using GAPDH. Error bars indicate the range of values obtained from two independent experiments

mRNA expression levels of different antiviral and ISGs in RAW264.7 and HEK293T cells in response to CP treatment were further investigated, and levels in CP-treated cells (1.0 μg/ml) were similar to positive control compared to non-treated cells (Fig. 5) (Primers are listed in Tables 3 and 4). In RAW264.7 cells, time-dependent up-regulated IFN-β, IL-6, TNF-α, ISG15, ISG56 and Mx-1 mRNA expression levels were observed in CP treated cells similar to the patterns of positive control (Fig. 5a, b). Moreover, most of the antiviral or inflammation related gene transcription levels were up-regulated in HEK293T cells (Fig. 5c). Especially, IFN-β, Mx1, GBP-1, IL-6, IL-8 and TNF-α mRNA expression levels at 12 hpt were increased by 15-, 14-, 6-, 20-, 12- and 9-fold, respectively. Additionally, extracts induced the transcription of ISGs, such as ISG-15 and ISG-56 (23- and 35-fold), respectively at 12 hpt compared to non-treated cells. The observed patterns were similar to the pattern of the rhIFN-β-treated control (Fig. 5c). As a result, CP has the capacity to up-regulate IFN-β, ISGs and various antiviral gene transcriptional levels and this activation may have a direct relationship with the antiviral abilities of the extract, which were observed in RAW264.7, HEK293T and HeLa cells.

**Table 3:** Mouse primer sets used to confirm mRNA expression

Gene	Primers	
	Forward	Reverse
IFN-β	5′-TCCAAGAAAGGACGAACATTCG-3′	5′-TGCGGACATCTCCCACGTCAA-3′
Mx1	5′-ACAAGCACAGGAAACCGTATCAG-3′	5′-AGGCAGTTTGGACCATCTTAGTG-3′
ISG-15	5′-CAATGGCCTGGGACCTAAA-3′	5′-CTTCTTCAGTTCTGACACCGTCAT-3′
ISG-56	5′-AGAGAACAGCTACCACCTTT-3′	5′-TGGACCTGCTCTGAGATTCT-3′
TNF-α	5′-AGCAAACCACCAAGTGGAGGA-3′	5′-GCTGGCACCACTAGTTGGTTGT-3′
IL-6	5′-TCCATCCAGTTGCCTTCTTGG-3′	5′-CCACGATTTCCCAGAGAACATG-3′
GAPDH	5′-TGACCACAGTCCATGCCATC-3′	5′-GACGGACACATTGGGGGTAG-3′

**Table 4:** Human primer sets used to confirm mRNA expression

Gene	Primers	
	Forward	Reverse
IFN-β	5′-CATCAACTATAAGCAGCTCCA-3′	5′-TTCAAGTGGAGAGCAGTTGAG-3′
Mx-1	5′-CCAAAGACACTTCCTCTC-3′	5′-CAGTGTGGTGGTTGTACT-3′
GBP-1	5′-AGAGATCACGGACTACAGAA-3′	5′-TCTGTGGACGTGTCATAGAT-3′
ISG-15	5′-GAGAGGCAGCGAACTCATCT-3′	5′-CTTCAGCTCTGACACCGACA-3′
ISG-56	5′AAGGCAGGCTGTCCGCTTA-3′	5′-TCCTGTCCTTCATCCTGAAGCT-3′
IL-8	5′-CTCTCTTGGCAGCCTTCCTGATT-3′	5′-AACTTCTCCACAACCCTCTGCAC-3′
IL-6	5′-CCACACAGACAGCCACTCACC-3′	5′-CTACATTTGCCGAAGAGCCCTC-3′
TNF-α	5′-ATGAGCACTGAAAGCAT-3′	5′-TCGACGGGGAGTCGAACT-3′
β-actia	5′-CCAACCGCGAGAAGATGACC-3′	5′-GATCTTCATGAGGTAGTCAGT-3′

### HPLC analysis of aqueous plant extract of Cortex Phellodendri, identification of berbrine and effects of berberine on virus replication

**Fig. 6:**
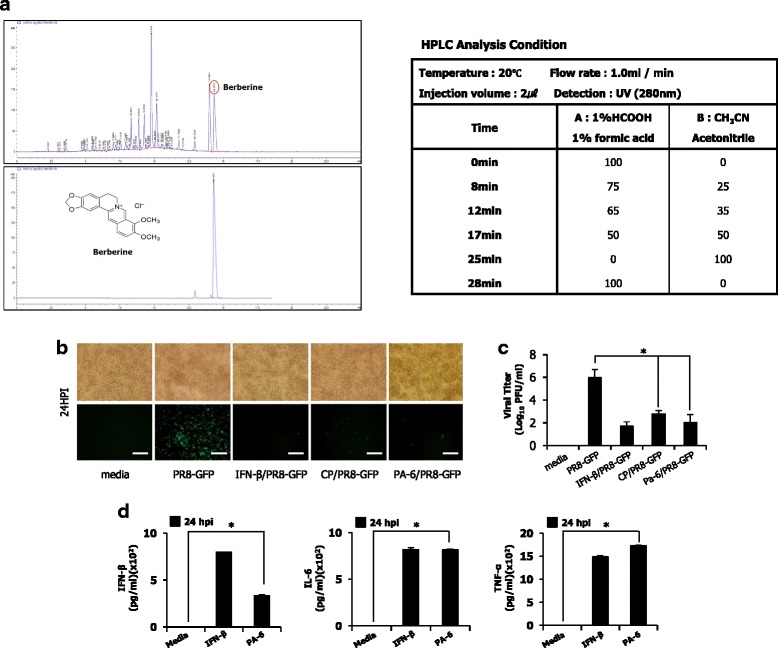
Chemical characterization of Cortex Phellodendri by HPLC analysis and effects of PA-6 on virus replication. **a** Chemical compounds in CP was analyzed by the reversed-phase HPLC. **b** RAW264.7 cells treated with media alone, 1.0 μg/ml CP, 10.0 μg/ml PA-6, or 1000 unit/ml recombinant mouse IFN-β at 12 h prior to infection with PR8-GFP (MOI = 1.0). GFP expression images were obtained at 12 and 24 hpi (200X magnification). **c** PR8-GFP titer was determined by standard plaque assay. **d** Secreted cytokines of murine IFN-β, IL-6 and TNF-α measured by ELISA. Tests were performed in triplicate. The data shows representative means ± SD. (*P < 0.05 indicates a significant difference between groups)

To investigate the critical components in CP, quantitative analysis was carried out by a reversed-phase HPLC method for the various fractions present in the CP and 6 fractions (PA-1, PA-2, PA-3, PA-4, PA-5 and PA-6) were successfully purified. Among 6 fractions, PA-6 (berberine) was detected at a wavelength and retention time of 280 nm & 12.885 min respectively, and 47.7 μg/mL of PA-6 present in 16.8 mg/mL (w/v) of CP (Fig. 6). The typical full-scan ESI-MS analyses of berberine and PA-6 is described in Additional file 1: Figure S1. These ESI-MS data compared to those reported in the literature [11–13], and PA-6 was identified as berberine. The antiviral effect of identified fractions (PA-1 to PA-6) was tested in RAW264.7 cells. An effective dose (10.0 μg/ml) was selected based on our preliminary experiments on the efficacy of purified fractions (Data not shown) and viral replication was observed with PR8-GFP in response to pre-treatment of various fractions. Remarkably, PA-6 inhibited the GFP expression compared to untreated groups and the viral titers were reduced by 3-fold at 24 hpi (Fig. 6c). Additionally, to find out whether PA-6 is the main compound in CP to stimulate cytokine secretion, IFN-β, IL-6 and TNF-α levels were measured after treatment of PA-6. Markedly, levels of IFN-β and cytokines secretion increased in RAW264.7 cells (Fig. 6d). These results suggest that berberine which is a major constituent of CP has the potential to inhibit the virus replication by inducing the antiviral state in the cells.

## Discussion

In the present study, the broad spectrum antiviral activity of medicinal herb C. Phellodendri both in vitro and in vivo was evaluated to suggest the rationale for its immune-modulatory and biological properties. C. Phellodendri has been reported as a well-known source for the variety of therapeutics with rare side effects after a dose [14], and in the tested cell lines, CP also did not show any significant cytotoxic effect. Moreover, the cell CC_50_ of CP was several magnitudes higher than the EC_50_ of tested viruses and the SI of the herb for various viruses indicate the higher safety margin of the extract for therapeutic and/or prophylactic purposes (Table 1).

In the present study, the CP inhibited the replication of PR8-GFP (Fig. 1a), VSV-GFP (Figs. 1b and 2a), NDV-GFP (Fig. 1c), HSV-GFP (Fig. 2b), H3-GFP (Fig. 2c) and EV-71 (Fig. 2d) in immune (RAW264.7) and epithelial (HEK293T and HeLa) cells. Interestingly, oral administration of CP not only increased the survival rate of mice subjected to lethal challenges with different influenza A virus subtypes including H1N1, H5N2, H7N3 and H9N2 but also led to rapid weight recovery (Fig. 3). Although CP-inoculated mice initially displayed little weight reduction, majority did not lose more than 25 % of their body weight. However, all of the mice in the control groups displayed more than 25 % losses within 9 dpi. Collectively, it suggest that CP is strong enough to inhibit the viral replication and promoted the survival of mice against lethal infections of diverse influenza A viruses.

Upon viral infection, host cells initially recognize the virus infection and rapidly evoke the antiviral innate immune responses including secretion of type I IFN and pro-inflammatory cytokines [15]. Secreted IFNs and cytokines induce an antiviral state which is important to protect the host cells against invading viruses, as induction of antiviral state in early time of virus infection is critical to control the spread and pathogenesis of viruses [16]. In this study, CP induces an antiviral state via the induction of type I IFN and pro-inflammatory cytokines in vitro. To elucidate the delineate features of CP in antiviral signaling, the effect of CP on the phosphorylation of IRF-3, p65, TBK1, STAT1, ERK and p38, the important regulatory molecules which present in the type I IFN [17–20] and NF-kB [21, 22] signaling pathways were studied and it shown to be up-regulated. Additionally, CP up-regulated the transcriptional levels of IFN-β, ISGs and various antiviral genes. These findings confirm that CP can induce the innate immune responses via type I IFN and NF-kB signaling for controlling viral replication.

In this study, murine macrophages (Raw264.7) were used to evaluate the antiviral effect of CP and also used epithelial cells such as HEK293T or HeLa cells which are very susceptible for viral replication. Recently, it is acknowledged that HEK293T [23] and HeLa [24] cells have less prominent pattern recognition receptors (PRRs), chiefly Toll-like receptors (TLRs). Thus it could postulate that CP may contain active components which can bind or penetrate the cell membrane and those active components may stimulate the cell surface PRRs, cytoplasmic PRRs or both and ultimately, can induce the antiviral immune responses in immune cell or epithelial cells. A bacterial endotoxin, LPS (or lipid A-associated protein) is a known immunomodulator and is often a contaminant in biological preparations. Therefore, it could speculate that macrophage-stimulating properties of CP might be due to contamination from bacterial endotoxin [25]. In this study, CP was tested with LPS using a LAL assay and was found only a trace amount of endotoxin (Fig. 4d), and antiviral function of CP was not due to LPS stimulation.

The constituents of CP are numerous and diverse, including aporphine alkaloids, protoberberine alkaloids, flavonoids, berberine, oxyberberine, palmatine, isovanillin, ferulic acid, limonin and various other molecules [4]. HPLC analysis was conducted to identify different active compounds present in the total aqueous fraction and found six major peaks (Fig. 6a), and six fractions (PA-1 to PA-6) which were successfully purified. Among six fractions, PA-6 was identified to have significant antiviral properties similar to a CP and later confirmed as berberine (Fig. 6c). Berberine has already been studied for its anticancer properties [26], antibacterial activity [27], antifungal activity [28] and antiviral activity [29]. Especially, it was reported that berberine inhibit influenza viral replication by reducing the viral neuraminidase activity, and reducing inflammatory responses which induced by influenza virus through inhibition of nitric oxide (NO) and repression of TNF-α and monocyte chemoattractant protein 1 (MCP-1) gene transcription [30]. Moreover, HB-13, a compound derived from berberine, also inhibits HSV-1 and HSV-2 activity [31, 32]. However, in the present study, the antiviral effects of berberine was demonstrated with more specific mechanism via type I IFN stimulation. Consequently, antiviral or immunomodulatory effects of CP could be due to the cumulative effect of berberine or various other unknown active compounds present in the extract.

Modern pharmacological researches have further confirmed that alkaloids of C. Phellodendri could be used for treating many diseases. Among them, relationship between mechanisms of antiviral effects and active compounds including berberine should be required further study. Furthermore, the question of how to address the need for both individualizing (the basis of TCM) and standardizing (the basis of modern pharmacology) the treatments with C. Phellodendri must be settled. Once these issues are resolved, the prospects will be exists for widespread use of C. Phellodendri as a safe, effective, and affordable form of healthcare.

## Conclusion

The current study propose that pre-treatment of CP has a broad spectrum of anti-viral functions against VSV-GFP, PR8-GFP, NDV-GFP, HSV-GFP, H3-GFP and EV-71 in vitro and against divergent influenza A subtypes of H1N1, H5N2, H7N3 and H9N2 in vivo mouse model. Moreover, our results describe a possible mode of CP antiviral activity via the induction of type I IFN or pro-inflammatory cytokines and the subsequent stimulation of the antiviral state in the host cell. Additionally, active components present in CP including berberine are important for the observed antiviral and immunomodulatory properties. Thus, the use of C. Phellodendri as an orally active antiviral agent, together with present vaccines or individually, has the potential to be an effective remedy in both humans and livestock for prophylaxis applications.

## Abbreviations

C. Phellodendri, Cortex Phellodendri; CC_50_, cytotoxicity concentration; CP, Cortex Phellodendri aqueous extract; CPE, cytopathic effects; dpi, days post-infection; EC_50_, effective concentration; ERK, extracellular signal-regulated kinases; ESI-MS, electrospray ionization mass spectrometry; EU, endotoxin unit; EV-71, enterovirus-71; GAPDH, glyceraldehyde 3-phosphate dehydrogenase; GBP-1, guanylate binding protein 1; GFP, green fluorescent protein; H3: coxsackie virus; HA, hemagglutination assay; hpi, hours post-infection; HPLC, high-performance liquid chromatography; HSV, herpes simplex virus; IFN, interferon; IL, interleukin; iNOS, inducible isoform nitric oxide synthases; IRF-3, interferon regulatory transcription factor 3; ISGs, interferon-stimulating genes; LAL, limulus amebocyte lysate; LC-MS, liquid chromatography-mass spectrometry: LPS, lipopolysaccharide; M-gene: matrix gene; MCP-1, monocyte chemoattractant protein 1; MLD_50_, 50 % minimum lethal dose; MOI, multiplicity of infection; Mx1, interferon-induced GTP-binding protein; NDV, newcastle disease virus; NF-kB, nuclear factor kappa-light-chain-enhancer of activated B cells; NO, nitric oxide; PA, purified fraction; PR8, influenza A virus; PRRs, pattern recognition receptors; qRT-PCR, quantitative real-time polymerase chain reaction; RBC, red blood cells; RBI: relative band intensity; rhIFN-β, recombinant human interferon-β; rmIFN-β, recombinant mouse interferon-β; RT-PCR, real-time polymerase chain reaction; SI, selection indexes; STAT1, signal transducer and activator of transcription 1; TBK1, TANK-binding kinase 1; TCID_50_, 50 % tissue culture infectious doses; TLRs, toll-like receptors; TNF-α, tumor necrosis factor-alpha; VSV, vesicular stomatitis virus

## Additional file

Additional file 1: Figure S1. Electrospray ionization mass spectra of PA-6 and berberine. The molecular weight identification of PA-6 (A) and berberine (B) was carried out by ESI-MS. (PPTX 145 kb)

## References

[1] Lemon SM, Mahmoud AA (2005). The threat of pandemic influenza: are we ready?. Biosecurity and bioterrorism : biodefense strategy, practice, and science.

[2] Arzt J, White WR, Thomsen BV, Brown CC (2010). Agricultural diseases on the move early in the third millennium. Vet. Pathol..

[3] Mukhtar M, Arshad M, Ahmad M, Pomerantz RJ, Wigdahl B, Parveen Z (2008). Antiviral potentials of medicinal plants. Virus Res..

[4] Xian YF, Mao QQ, Ip SP, Lin ZX (2011). Che CT Comparison on the anti-inflammatory effect of Cortex Phellodendri Chinensis and Cortex Phellodendri Amurensis in 12-O-tetradecanoyl-phorbol-13-acetate-induced ear edema in mice. J. Ethnopharm..

[5] Magadula S (2010). Cytotoxic and anti-HIV activities of some Tanzanian Garcinia species. Tanzania J Health Res.

[6] Strober W. Trypan blue exclusion test of cell viability. Current protocols in immunology. 2001;Appendix 3:Appendix 3B. 10.1002/0471142735.ima03bs21 18432654

[7] Moon HJ, Lee JS, Choi YK, Park JY, Talactac MR, Chowdhury MY, Poo H, Sung MH, Lee JH, Jung JU (2012). Induction of type I interferon by high-molecular poly-gamma-glutamate protects B6.A2G-Mx1 mice against influenza A virus. Antiviral Res.

[8] Seal BS, King DJ, Bennett JD (1995). Characterization of Newcastle disease virus isolates by reverse transcription PCR coupled to direct nucleotide sequencing and development of sequence database for pathotype prediction and molecular epidemiological analysis. J. clin. microb..

[9] Reed JR, Leon RP, Hall MK, Schwertfeger KL (2009). Interleukin-1beta and fibroblast growth factor receptor 1 cooperate to induce cyclooxygenase-2 during early mammary tumourigenesis. BCR.

[10] Zhao G, Lin Y, Du L, Guan J, Sun S, Sui H, Kou Z, Chan CC, Guo Y, Jiang S (2010). An M2e-based multiple antigenic peptide vaccine protects mice from lethal challenge with divergent H5N1 influenza viruses. Virol..

[11] Yu S, Pang X, Deng Y, Liu L, Liang Y, Liu X, Xie L, Wang G, Wang X (2007). A sensitive and specific liquid chromatography mass spectrometry method for simultaneous determination of berberine, palmatine, coptisine, epiberberine and jatrorrhizine from Coptidis Rhizoma in rat plasma. Int. J. Mass Spectrom..

[12] Shuang-lai Z, Sheng-shan D, Xin-ru L, Run-hui L, Wei-dong Z, Hong-lin H, Yi Z, Yao-hua H, Shu-ping W (2011). Qualitative and Quantitative Analysis of Alkaloids in Cortex Phellodendri by HPLC-ESI-MS_MS and HPLC-DAD. Chem. Res. Chinese Universities.

[13] Yan R, Wang Y, Liu Y, Di X (2012). Comparative Pharmacokinetics of Berberine, Palmatine and Jatrorrhizine in Rat Plasma after Oral Administration of Rhizoma coptidis and Zuojinwan Using Liquid Chromatography-Tandem Mass Spectrometry. Iranian J. Pharm. Res..

[14] Huang CY, Tsai YT, Lai JN, Hsu FL (2013). Prescription pattern of chinese herbal products for diabetes mellitus in taiwan: a population-based study. Evid.-Based Compl. Alt. Med..

[15] Takeuchi O, Akira S (2007). Recognition of viruses by innate immunity. Immuno. Rev..

[16] Boasso A, Type I (2013). Interferon at the Interface of Antiviral Immunity and Immune Regulation: The Curious Case of HIV-1. Scientifica.

[17] Sato M, Suemori H, Hata N, Asagiri M, Ogasawara K, Nakao K, Nakaya T, Katsuki M, Noguchi S, Tanaka N (2000). Distinct and essential roles of transcription factors IRF-3 and IRF-7 in response to viruses for IFN-alpha/beta gene induction. Immunity.

[18] Zhu X, Wen Z, Xu LZ, Darnell JE (1997). Stat1 serine phosphorylation occurs independently of tyrosine phosphorylation and requires an activated Jak2 kinase. Mol. Cell. Biol..

[19] Goh KC, Haque SJ, Williams BR (1999). p38 MAP kinase is required for STAT1 serine phosphorylation and transcriptional activation induced by interferons. EMBO.

[20] David M, Petricoin E, Benjamin C, Pine R, Weber MJ, Larner AC (1995). Requirement for MAP kinase (ERK2) activity in interferon alpha- and interferon beta-stimulated gene expression through STAT proteins. Science.

[21] Pomerantz JL, Baltimore D (1999). NF-kappaB activation by a signaling complex containing TRAF2, TANK and TBK1, a novel IKK-related kinase. EMBO.

[22] Zhong H, Voll RE, Ghosh S (1998). Phosphorylation of NF-kappa B p65 by PKA stimulates transcriptional activity by promoting a novel bivalent interaction with the coactivator CBP/p300. Mol. cell.

[23] Graham KL, Lee LY, Higgins JP, Steinman L, Utz PJ, Ho PP (2010). Treatment with a toll-like receptor inhibitory GpG oligonucleotide delays and attenuates lupus nephritis in NZB/W mice. Autoimmunity.

[24] Kolli D, Velayutham TS, Casola A (2013). Host-Viral Interactions: Role of Pattern Recognition Receptors (PRRs) in Human Pneumovirus Infections. Pathog.

[25] Magalhaes PO, Lopes AM, Mazzola PG, Rangel-Yagui C, Penna TC, Pessoa A (2007). Methods of endotoxin removal from biological preparations: A review. J. Pharm. Sci.

[26] Sun Y, Xun K, Wang Y, Chen X (2009). A systematic review of the anticancer properties of berberine, a natural product from Chinese herbs. Anti-cancer drugs.

[27] Iwasa K, Kamigauchi M, Ueki M, Taniguchi M (1996). Antibacterial activity and structure-activity relationships of berberine analogs. Euro. J. Med. Chem.

[28] Iwazaki RS, Endo EH, Ueda-Nakamura T, Nakamura CV, Garcia LB, Filho BP (2010). In vitro antifungal activity of the berberine and its synergism with fluconazole. Anton. Leeuw..

[29] Hayashi K, Minoda K, Nagaoka Y, Hayashi T, Uesato S (2007). Antiviral activity of berberine and related compounds against human cytomegalovirus. Bioorganic & medicinal chemistry letters.

[30] Wu Y, Li JQ, Kim YJ, Wu J, Wang Q, Hao Y (2011). In vivo and in vitro antiviral effects of berberine on influenza virus. Chin. J. Integr. Med.

[31] Wu JB, Zheng JR, Lin Z, Li XY, Cui PG: Research of antiviral mechanisms of action of berberine derivate HB-13 against herpes simplex virus type 2. Chin. J. Antibiot. 2009; 34:376–79.

[32] Wu JB, Zheng JR, Lin Z, Li XY, Cui PG: In vitro antiviral activity of a berberine derivant HB-13 against herpes simplex virus. Chin. J. Dermatol. 2007; 40:671-73.

