# Supplementary material for: Inhibitory effects of an aqueous extract from Cortex Phellodendri on the growth and replication of broad-spectrum of viruses in vitro and in vivo
Source: BMC Complement Altern Med. 2016 Aug 2;16:265. doi: 10.1186/s12906-016-1206-x (PMC4970287; doi:10.1186/s12906-016-1206-x)
Supplement: Additional file 1: Figure S1. — Electrospray ionization mass spectra of PA-6 and berberine. The molecular weight identification of PA-6 (A) and berberine (B) was carried out by ESI-MS. (PPTX 145 kb) [file 12906_2016_1206_MOESM1_ESM.pptx]

## Slide 1
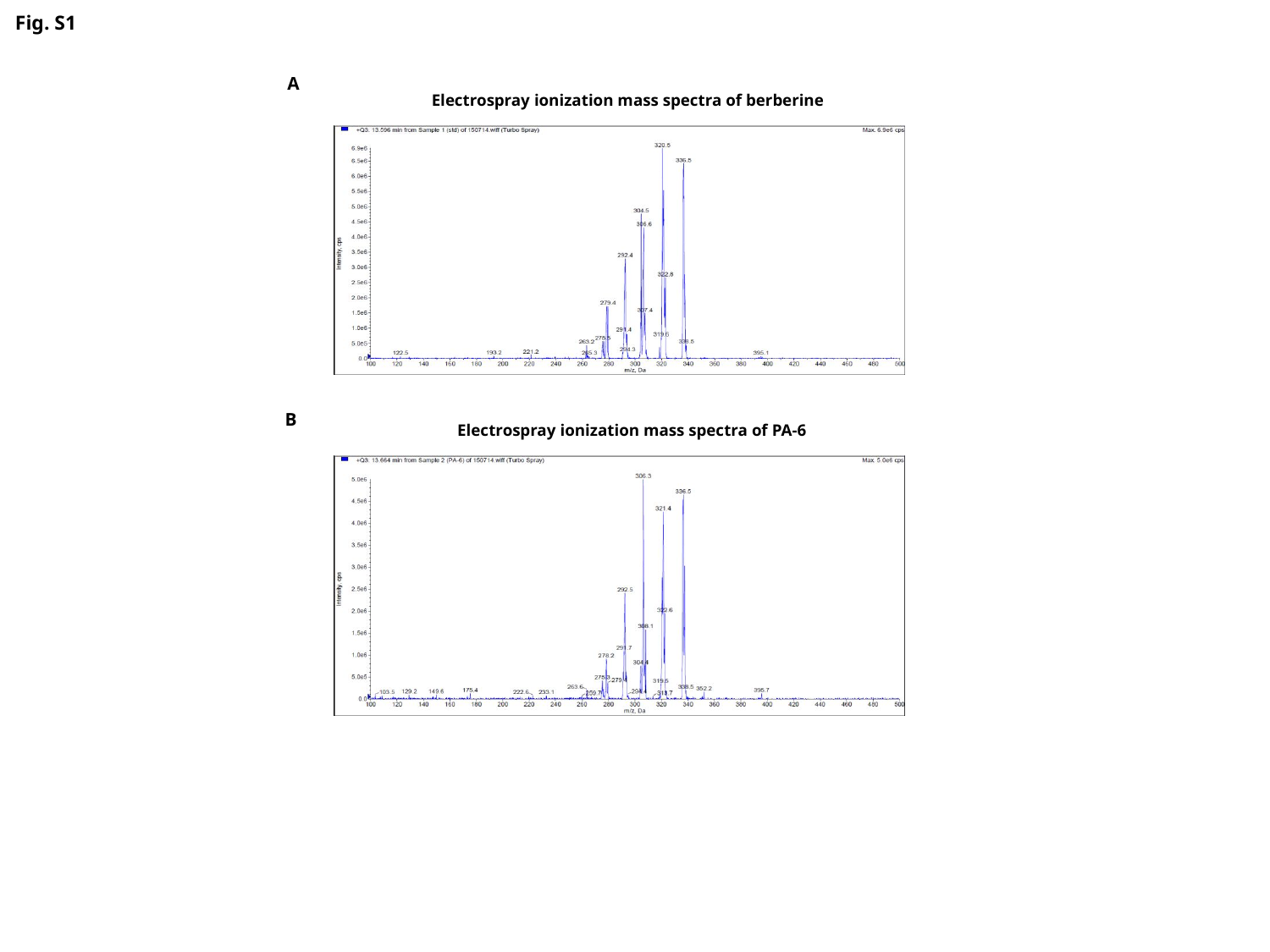

Fig. S1
A
Electrospray ionization mass spectra of berberine
B
Electrospray ionization mass spectra of PA-6
